# Neural Networks Recapitulation by Cancer Cells Promotes Disease Progression: A Novel Role of p73 Isoforms in Cancer-Neuronal Crosstalk

**DOI:** 10.3390/cancers12123789

**Published:** 2020-12-16

**Authors:** Stella Logotheti, Stephan Marquardt, Christin Richter, Renée Sophie Hain, Nico Murr, Işıl Takan, Athanasia Pavlopoulou, Brigitte M. Pützer

**Affiliations:** 1 Institute of Experimental Gene Therapy and Cancer Research, Rostock University Medical Center, 18057 Rostock, Germany; stephan.marquardt@med.uni-rostock.de (S.M.); christin.richter@med.uni-rostock.de (C.R.); renee.hain@uni-rostock.de (R.S.H.); nico.murr@med.uni-rostock.de (N.M.); 2 Izmir Biomedicine and Genome Center (IBG), 35340 Balcova, Izmir, Turkey; isil.takan@msfr.ibg.edu.tr (I.T.); athanasia.pavlopoulou@ibg.edu.tr (A.P.); 3 Izmir International Biomedicine and Genome Institute, Dokuz Eylül University, 35340 Balcova, Izmir, Turkey; 4 Department Life, Light & Matter, University of Rostock, 18059 Rostock, Germany

**Keywords:** cancer–neural crosstalk, neoneurogenesis, perineural invasion, metastasis, melanoma, p73, antineurogenic therapies

## Abstract

**Simple Summary:**

Cancer is initiated by alterations in specific genes. However, at late stages, cancer cells become metastatic not necessarily through continuous accumulation of additional mutations, but by hijacking programs of normal embryonic development and reactivating them in an unusual place, at the wrong time. Here, we applied computational and experimental approaches to show that these malignant reactivations include genes that are crucial for the development and function of the nervous system. We use the paradigm of melanoma transition from less invasive to highly aggressive stages in order to show that major players of metastasis, such as TP73 gene products, are implicated in this process. This work provides evidence for interactions between cancer cells and the neuronal system, which may have important future implications for metastasis prevention and cancer management.

**Abstract:**

Mechanisms governing tumor progression differ from those of initiation. One enigmatic prometastatic process is the recapitulation of pathways of neural plasticity in aggressive stages. Cancer and neuronal cells develop reciprocal interactions via mutual production and secretion of neuronal growth factors, neurothrophins and/or axon guidance molecules in the tumor microenvironment. Understanding cancer types where this process is active, as well as the drivers, markers and underlying mechanisms, has great significance for blocking tumor progression and improving patient survival. By applying computational and systemic approaches, in combination with experimental validations, we provide compelling evidence that genes involved in neuronal development, differentiation and function are reactivated in tumors and predict poor patient outcomes across various cancers. Across cancers, they co-opt genes essential for the development of distinct anatomical parts of the nervous system, with a frequent preference for cerebral cortex and neural crest-derived enteric nerves. Additionally, we show that p73, a transcription factor with a dual role in neuronal development and cancer, simultaneously induces neurodifferentiation and stemness markers during melanoma progression. Our data yield the basis for elucidating driving forces of the nerve–tumor cell crosstalk and highlight p73 as a promising regulator of cancer neurobiology.

## 1. Introduction

Despite current advances in diagnosis and treatment of primary tumors, the management of aggressive forms remains a still unmet challenge [1]. During cancer progression, cells acquire a set of key adaptations or hallmarks that increase the likelihood to obtain “metastatic potential”. This covers any combination of cancer phenotypes that enable metastatic dissemination, including motility and immune evasion as well as the ability to survive in the circulation and proliferate at distant sites, and is further shaped by changes in the tumor microenvironment (TME) [2]. Understanding the distinct events that govern tumor progression is essential for rational design of anti-metastatic strategies.

Recent studies indicate only rare metastasis-specific mutations, while activations of multiple transcriptional modules are a common theme [3]. It was recently proposed that metastatic transcriptional programs arise from de novo combinatorial activation of multiple distinct and developmentally distant transcriptional modules [3]. In line with this, accumulating data indicate that re-expression of genes and/or pathways in the wrong place and at the wrong time offers selective advantages towards acquisition of the metastatic potential. In particular, genes that are normally expressed in anatomical sites other than the site of tumor growth, such as tissue-restricted genes which are normally silent in most tissues [4,5] or genetic programs activated in different developmental/differentiation stages, such as programs of embryonic development [6,7] and human placentation [8], are reactivated “off-context” in metastatic cells. A universally recognized example is the reactivation of the epithelial–mesenchymal transition (EMT) that controls the neural crest, which gives rise to a variety of vertebrate structures, across several aggressive cancer types (reviewed in [9]). These examples support inappropriate reactivation of genes or pathways, and their “co-option”, as a recurrent mechanistic pattern during tumor progression. The term co-option, as used herein, refers to the process through which a biological function/structure that fulfills a certain role within one context may be alternatively used in another context to create a novel function.

An intriguing case of co-option is that cancer cells may recapitulate features of neuronal cells and reactivate mechanisms of neural differentiation and/or plasticity to achieve progression. Accumulating evidence supports a capacity of tumors to stimulate their own innervation during cancer progression. The infiltration of tumors by growing nerves is termed as neoneurogenesis [10]. It was recently unveiled that neural progenitors from the subventricular zone of the central nervous system (CNS) break the blood–brain barrier and infiltrate prostate tumors, in which they initiate neurogenesis [11]. A tumor is able to produce and excrete neuronal growth factors, neurothrophins and/or axon guidance molecules [12,13] that have the potential to modulate the TME, induce formation of new nerves and eventually influence the course of disease aggressiveness. Neoneurogenesis is essentially distinct from perineural invasion (PNI), which refers to tumor cells invading into already existing nerves along the perineural space [13]. In case of PNI, not only the nerve fibers create a physical scaffold that facilitates the migration of cancer cells, but also the perineural space is enriched with neuronal factors that create a protective niche for tumor cells, supporting their survival and proliferation. This “fatal attraction” between cancer and neuronal cells appears to be reciprocal. Both cancer cells and nerve fibers secrete factors that favor rapid growth of both, making the neural–epithelial interaction a mutually beneficial process [14]. In addition, cancer cells themselves may acquire brain-like properties as an adaptation for brain colonization. Indeed, breast-to-brain metastatic tissue and cells display phenotypes and metabolism similar to that of neuronal cells [15]. Moreover, it was described that malignant melanoma exhibits cytological characteristics of nerve cells [16] and neurons generated from cancer stem cells (CSC) support cancer progression [17].

The CNS emerges as an understudied parameter of tumor initiation and progression. PNI is a metastasis-related process occurring in highly innervated regions of the body, such as the prostate, head and neck, pancreas, salivary glands, liver and colon [18]. Far less understood is neoneurogenesis [14], and even more unexplored is the putative tendency of tumors to transform themselves to brain-like tissues [15], which have been described for prostate and breast cancer, respectively. Current data pinpoint towards two plausible mechanistic scenarios that might govern cancer cell–nervous system interactions either separately or in combination: (a) cancer cells themselves transdifferentiate into cells reminiscent of neural cells or tissues (“stealing identity”); and (b) tumors deterministically reactivate selected neuronal signaling pathways in order to influence the behavior of adjacent neuronal cells and create a positive tumor microenvironment (“hijacking signaling”). It is currently not known if this cancer–neuronal crosstalk has a more universal character and a potential cancer type-independent nature. Therefore, it is essential to characterize not only the cancer types whose metastatic transformation is orchestrated by recapitulation of neuronal attributes, but also the drivers and markers of this metastasis-promoting process. Herein, we shed light on the neuronal processes that are co-opted across cancer types, by integrating data from 35 human cancers, developmental phenotypes of knockout mice and differential gene expression profiles from distinct stages of differentiation of induced pluripotent stem cells to neurons. In addition, we provide experimental evidence that p73, a transcription factor with a dual role in neuronal development and cancer, is an emerging regulator of the interplay between the nervous system and cancer progression.

## 2. Results

### 2.1. Phenotype-Driven Identification of Genes Essential for Normal Nervous System Development and Neurological Function

**Figure 1:**
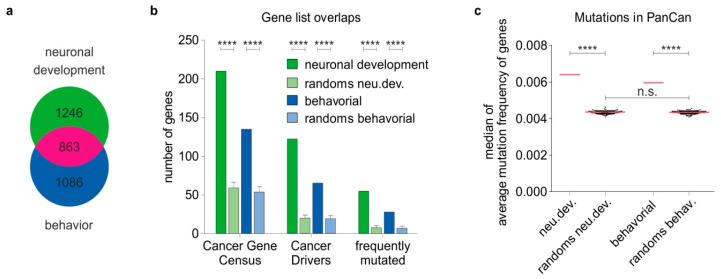
Nervous system-related genes undergo frequent gene mutations across cancer types. (**a**) Nervous system-related genes were defined as genes where the knockout of which in mice leads to abnormal phenotypes of neuronal development or behavior/neurological function. (**b**) Left: a significant enrichment of Cancer Gene Census factors was observed among the nervous system-related genes relative to the random control lists. Middle: the same analysis in a group of 127 significantly mutated genes which have been identified as oncogenesis drivers across 12 major cancer types, whereby most tumors bear two to six of these mutations [19]. Right: the same tendency was observed when nervous system-related genes were juxtaposed to a list of 299 driver genes and mutations identified from a comprehensive PanCancer and PanSoftware analysis spanning 9423 tumor exomes and using 26 computational tools [20]. (**c**) The median over all genes of the list of the average mutation frequency of nervous system-related genes is significantly higher in the PanCan cohort versus genes of the random lists. This entails a higher number of patients exhibiting mutations in nervous system-related genes. Z-test was used for statistical analyses; **** *p* < 0.0001, n.s.: non-significant.

Links between the nervous system and metastatic potential can be plausibly inferred by the reactivation of genes supporting neuronal development and/or neurological function in aggressive cancer cells. For a comprehensive identification of genes that are indispensable for these processes, we screened the Mouse Genome Informatics (MGI) database for genes where the knockout of which causes defects during development of the nervous system or to neurological disorders, using relevant terms (see Materials and Methods and Tables S1 and S2). By matching each gene with the corresponding nervous system—or behavior-affected phenotypes—we obtained a list comprising all protein-coding genes (PCGs) whose knockout in mice: (a) affects embryonic development of the nervous system, leading to malformations of neural system components and/or abnormal neuron cell number or morphology (thereafter referred to as the neu.dev gene list) (Table S1), and (b) leads to neurological and behavioral disorders, such as ataxia, seizures, defects in learning and memory and abnormal mouse behavior (thereafter called the behavioral gene list) (Table S2). Overall, we generated a list of 2119 neu.dev PCGs and 1954 behavioral PCGs. Highly conserved human homologues exist for 2109 and 1949 of them, respectively (Tables S1 and S2). We found that 1246 are specific for neuronal development and 1086 for neurological function, while 863 genes are involved in both processes (Figure 1a). For clarity reasons, we collectively termed those genes that, based on their mouse phenotypes, appear to be essential for normal nervous system development and function, such as nervous system-related genes. We proceeded to unravel the associations of these genes with cancer.

### 2.2. Nervous System-Related Genes Are Frequently Mutated in Cancer Patient Tumors and Enriched in Cancer Driver Genes

We hypothesized that one route of reactivation of nervous system-related genes in the context of cancer includes, but is not limited to, genetic mutations. Hence, we initially sought to explore whether neu.dev and behavioral genes are associated with key oncogenic drivers. In this regard, we juxtaposed these genes with distinct lists of well-characterized cancer driver genes and driver mutations and determined their enrichment among the nervous system-related genes. To ensure that the association is non-random, we compared each of the neu.dev and behavioral lists with 100 control lists, whereby each control list encompasses an equal number of random, unrelated genes (2109 for neu.dev and 1949 for behavioral) (Table S3). First, we juxtaposed neu.dev and behavioral genes to the Cancer Gene Census list of gene mutations causally implicated in cancer and found a significant enrichment of oncogenic factors among both neu.dev and behavioral genes relative to their corresponding controls. Ten percent (210 neu.dev genes) and 6.9% (135 behavioral genes) were oncogenic drivers versus an expected by-chance proportion 3.6% (721 of 19,943 coding genes from GENCODEv32). These percentages were also higher compared to the control lists for each of the neu.dev or behavioral lists (2.76 ± 0.36% and 2.77 ± 0.35%, respectively, *p* < 0.0001 1.0 × 10^−13^, Figure 1b). We observed a consistent tendency, upon comparison of the nervous system-related genes versus a group of 127 significantly mutated genes which have been recently identified as oncogenesis drivers across 12 major cancer types, whereby most tumors bear two to six of these mutations [19]. A significant enrichment of 55 neu.dev and 28 behavioral genes (2.62% and 1.44% compared to expected 0.64% by chance) was detectable in relation to the control groups with 0.33 ± 0.13% and 0.36 ± 0.14% (*p* < 0.0001, Figure 1b). In a similar manner, comparison of the nervous system-related genes with 299 cancer driver genes and driver mutations identified by a comprehensive PanCancer and PanSoftware analysis spanning 9423 tumor exomes and using 26 computational tools [20] revealed that neu.dev genes constitute 5.80% (122 genes) and behavioral genes 3.33% (65 genes) of the identified cancer drivers versus 0.90 ± 0.19% and 0.97 ± 0.20% of their corresponding controls or 1.54% by chance (*p* < 0.0001). Subsequently, we examined if nervous system-related genes undergo frequent genetic alterations among cancer patients, using gene mutation data from the PanCan cohort. We determined the percentage of patients bearing mutations in the neu.dev and behavioral genes to all patients in the TCGA cancer atlas and ascertained that, compared to the random control lists, a higher percentage of cancer patients exhibit mutations in neu.dev and behavioral genes (Figure 1c). Overall nervous system-related genes exhibit a non-stochastic tendency to undergo frequent mutations in tumors, entailing genetic alterations on cancer driver genes.

### 2.3. Nervous System-Related Genes Are Preferentially Upregulated in Aggressive Stages and Predict Patient Outcomes in a Cancer Type-Dependent Manner

**Figure 2:**
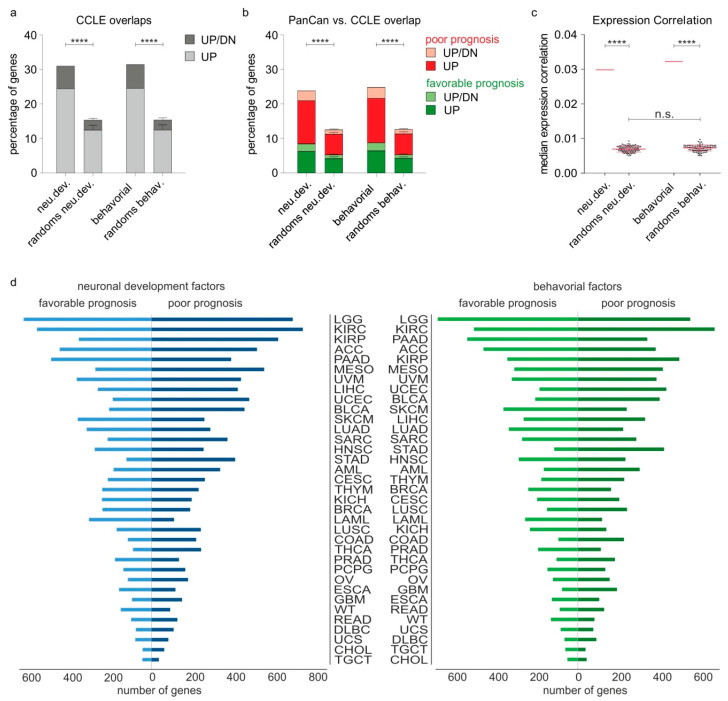
Nervous system-related gene transcripts are upregulated in invasive stages and predict clinical outcomes in a cancer type-dependent manner. (**a**) Transcripts of both neu.dev and behavioral genes are upregulated in the highly invasive versus the less invasive CCLE cell lines, in a percentage higher than the corresponding percentages of the control lists. Light gray depicts the genes that are upregulated across all cancer types, while the dark gray depicts the genes that show cancer type-dependent upregulation. (**b**) Cox regression analysis of the nervous system-related genes that are upregulated in invasive cell lines of CCLE on the survival of the TCGA PanCan cohort patients. Genes that correlate with poor prognosis and are upregulated in aggressive states of CCLE are depicted in red (UP, exclusively upregulated in all cancer types) or pink (UP/DN, upregulated in some as well as downregulated in other cancer types). The ones that correlate with favorable prognostic factors are depicted in dark (UP) or light green (UP/DN). (**c**) Shown are the medians of expression correlation (MEC) in PanCan within each gene list of the neu.dev, the behavioral and the respective 100 random gene lists (mean with SD pink lines). MEC are significantly higher among neu.dev and behavioral genes compared to their respective random lists. (**d**) Survival analysis for neu.dev (left, blue) and behavioral (right, green) genes across different TCGA cancers. The prognostic potential of each nervous system-related gene depends on the cancer type. In the majority of cancer types, more nervous system-related genes are associated with poor and less with favorable prognosis. Z-test was used for statistical analyses; **** *p* < 0.0001, n.s.: non-significant.

Our finding that nervous system-related genes bear frequent genetic alterations in tumors suggests that their transcriptional activity might be consequently affected. Enhanced transcription of nervous system-related genes could lead to manifestation of neuronal characteristics in tumors and provide possible molecular links between cancer progression and nervous system development and/or function. Hence, we checked if the transcripts of nervous system-related genes are upregulated in high- versus low-invasive cancer stages, using a multiomics approach that integrated high-throughput data derived from cell lines and tumor transcriptomes of cancer patients. To this end, we classified all cell lines of the Cancer Cell Line Encyclopedia (CCLE) [21], which includes gene expression data from 1076 cell lines, across 18 common cancer types, into highly invasive and less invasive on the basis of the expression levels of reliable markers for EMT and tumor progression such as N-cadherin, E-cadherin, Vimentin, ZEB1 and SNAI1 (Table S4) [22], and subsequently verified if neu.dev and/or behavioral genes are upregulated according to the invasive capacity of the tumor cells. For the neu.dev group, we found that 513 (24.3% of neu.dev) genes are significantly upregulated in highly invasive cells across cancer types, while for 138 (6.5%), upregulation depends on the cancer type. Similarly, for the behavioral list, we noticed that 478 (24.4% of behavioral) genes were strongly expressed in high-invasive stages and for 135 (6.9%), upregulation is cancer-dependent. On the contrary, the number of neu.dev and behavioral genes that are downregulated in aggressive cell lines is significantly less, 393 (18.6%) and 391 (20%), respectively. These percentages were significantly higher (*p* < 0.0001) and significantly biased towards upregulated genes (*p* < 0.01) compared to their corresponding controls, thus indicating a non-stochastic tendency for enhanced expression of nervous system-related gene transcripts in highly invasive cancer cells (Figure 2a).

To examine the clinical relevance of these findings, we checked if nervous system-related gene transcripts that are enhanced in invasive states are also associated with unfavorable patient outcomes. Hence, we meta-analyzed gene expression data in 35 different cancer types from the PanCan TCGA cohort [23]. Cox regression analysis revealed 321 neu.dev and 313 behavioral genes that are both upregulated in high-aggressive tumors in the CCLE and associated with poor patient outcomes in the PanCancer clinical data resource, whereas the number of genes whose downregulation is associated with poor prognosis is significantly lower (169 and 152) (Figure 2b and Table S5). Expression correlation analysis showed that a large fraction of both neu.dev and behavioral genes tend to be co-expressed in tumors of PanCan cohort patients, implying a tendency to preserve their functional connections and interplay in the context of cancer (Figure 2c). In sum, nervous system-related gene transcripts that are frequently upregulated upon invasiveness predict poor patient outcome.

Furthermore, we conducted Cox regression analysis to estimate the effect of each nervous system-related gene on the prognosis of individual cancer types. The results are demonstrated in Figure 2d and in Table S6. Based on the number of neu.dev or behavioral genes whose expression in cancer cells associates, either positively or negatively, with patient survival, the most affected cancer types are: LGG, KIRC, KIRP, ACC, PAAD, MESO, UVM, LIHC, UCEC, BLCA, SKCM, LUAD, SARC, HNSC, STAD and AML. The same cancer types are comparably affected by either neu.dev or behavioral genes. Moreover, up to 212 neu.dev and 148 behavioral genes are associated with poor patient outcomes in a minimum of 25% (9/35) of the examined cancer types, and a single gene can negatively affect the prognosis of up to 17 (median = 5.0 ± 2.7 for neu.dev and 4.0 ± 2.5 for behavioral genes, respectively) cancer types. No single gene was universally associated with the prognosis of all 35 cancers. Rather, the effect of each individual nervous system-related gene is cancer type-dependent. However, several nervous system-related genes were identified as common poor prognostic factors in 10 to 17 cancer types and thus hold a potential as more generalized indicators of neuronal co-options in the context of cancer progression (Table S6). These results underscore a link between deregulated transcription of nervous system-related genes and cancer progression.

### 2.4. Different Cancer Types Co-Opt Genes Essential for the Development of Distinct Anatomical Parts of the Nervous System

**Figure 3:**
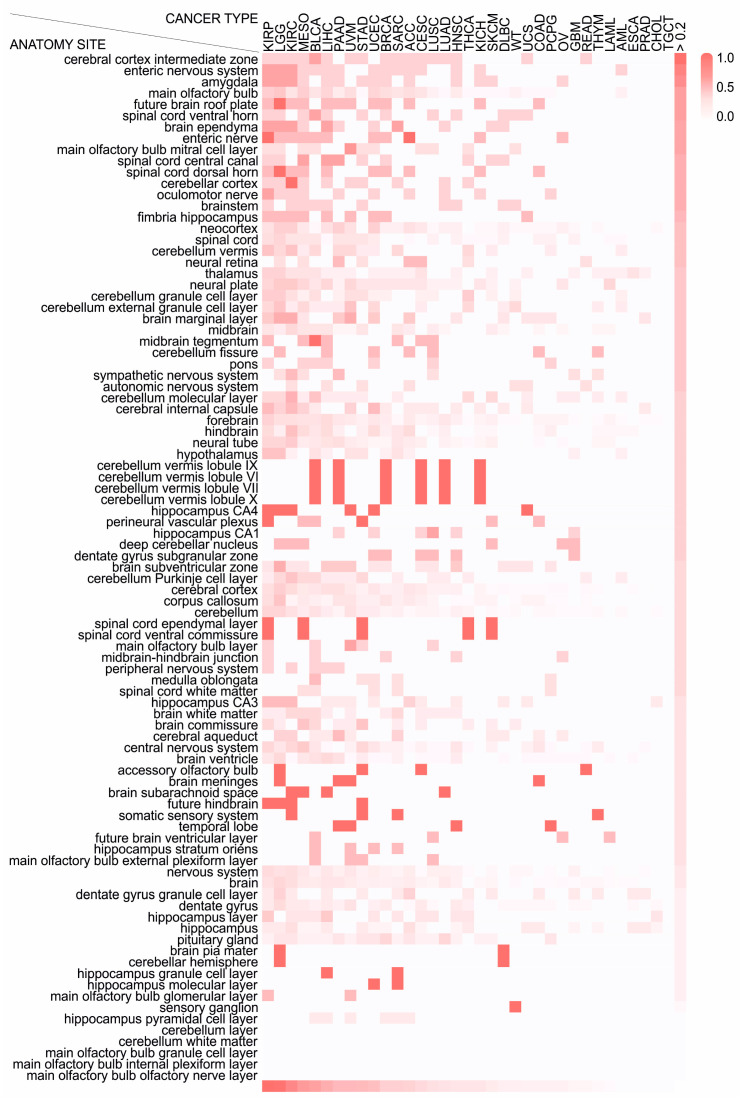
Genes indispensable for the development of distinct anatomical regions of the nervous system are hijacked by specific cancer types. A heatmap of anatomy sites that are enriched for neu.dev and behavioral genes that correlate with poor prognosis per cancer type. The color corresponds to the enrichment of each anatomic site (see legend).

The nervous system encompassses several contiguous anatomical regions which are responsible for many different and separate functions. CNS and the PNS both contribute to the same functions, but those are controlled by different regions of the brain or different ganglia in the periphery. These structures control different functions and present distinct patterns of gene expression that reflect their developmental history [24]. Thus, we sought to examine whether the nervous system-related genes that have an impact on patient prognosis are particularly associated with the development of specific regions of the CNS and/or PNS. To address this issue, using the information from Mammalian Phenotype (MP)-Mouse Developmental Anatomy (EMAPA) Mappings of the MGI database, we specified which anatomical structures are impaired upon knockout of neu.dev genes (Tables S1 and S2) and estimated if there is an enrichment for specific structures among those that were identified as poor prognostic factors across cancer types. First, we enlisted all anatomical regions where the development of which is impaired by knockdown of at least one neu.dev gene. Then, we allocated neu.dev genes according to the anatomical regions they affect and estimated the number of poor prognostic factors for each cancer type, per anatomical region. The enrichment was estimated as the number of poor prognostic factors associated with each anatomical region divided by the number of the neu.dev genes that are essential for this region. We found that genes indispensable for the normal development of distinct structures lead to poor prognosis upon their reactivation in the cancer cell context, and that the CNS anatomical structures that are usurped depend on cancer type. For instance, genes with a detrimental effect on 10–17 cancer types are essential for the development of the cerebral cortex intermediate zone, the enteric nervous system, the amygdala, the main olfactory bulb, the future brain roof plate, the spinal cord, the brain ependyma, the cerebellar cortex, the oculomotor nerve and the brainstem (Figure 3). Our findings clearly imply that tumors hijack specific anatomical regions of the nervous system to orchestrate their progression to aggressive stages, while the top affected areas are the neural tube-derived cerebral cortex intermediate zone and the neural crest-derived enteric nervous system.

### 2.5. Genes Involved in Several Stages of Neurogenesis and Neuronal Differentiation are Reactivated during Cancer Progression

New neurons originate from multipotent neural stem cells. Normally, neurogenesis is active in the developing embryonic brain, as well as in specific brain regions in adult mammals, mainly the subventricular zone (SVZ) of the lateral ventricles and the subgranular zone (SGZ) in the hippocampus. During this process, the pluripotent embryonic stem cells (ESCs) give rise to the multipotent neural stem cells (NSCs, or neural progenitors), which reside at the sites of neurogenesis and differentiate into neurons and glial cells in response to specific cues. Studies of the events that drive neurogenesis and neurodifferentiation have been experimentally facilitated by the development of the induced pluripotent cell (iPSC) technology, a revolutionary method developed by Takahashi and Yamanaka, to obtain stem cells from adult somatic cells. According to this technology, normal somatic, differentiated cells can be dedifferentiated to pluripotent stem cells that closely resemble embryonic stem cells through manipulation of four nodal factors (Oct4, Klf4, c-Myc, Sox2). Induced pluripotent stem cells are self-renewing and, upon appropriate and controlled reprogramming, can differentiate into specific cell types. Similar to ESCs, human iPSCs can give rise to neuronal lineage cells, such as neurons, astrocytes and oligodendrocytes, when exposed to the timely and coordinated function of regulatory factors such as BMP, Wnt and FGF. Overall, differentiation of iPSCs to neuronal cells in vitro largely simulates the process of neural induction and specification of stem cells, thereby providing insights on the molecular events that control each of the early, intermediate or late stages of neurogenesis and neurodifferentiation [25].

**Figure 4:**
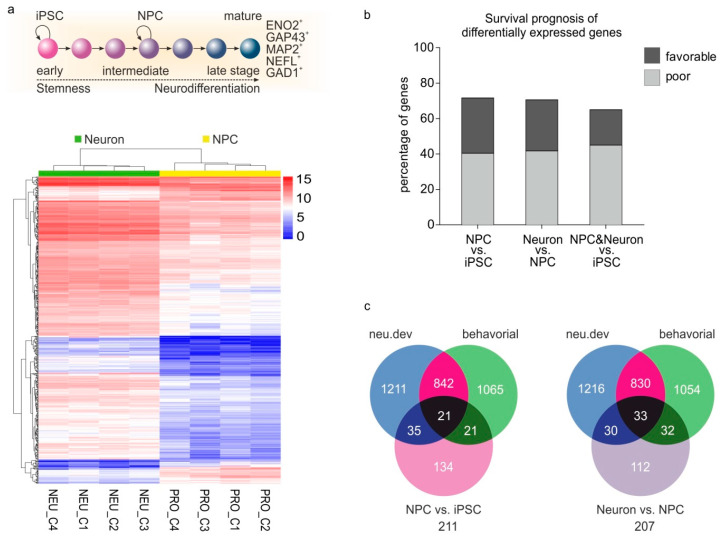
Genes involved in early and late stages of neuronal differentiation are co-opted during cancer progression. (**a**) Top: Scheme of differentiation from iPSCs to NPC and mature neurons. Markers of mature neurons which were used for the differential gene expression analysis are indicated. Bottom: Representative heatmap of differential gene expression in neurons versus NPCs for the study GSE135287. (**b**) Percentage of the differentially expressed genes between the indicated states of differentiation that have poor or favorable prognosis on patient outcomes in PanCancer. (**c**) Venn diagrams showing the overlaps of neu.dev and behavioral factors with the differentially expressed genes in NPC versus iPSC (left) and Neuron versus NPC (right). iPSC: induced pluripotent stem cell; NPC: neural progenitor cell.

Subsequently, we questioned whether cancer cells “usurp” genes that are active over the whole range of neurodifferentiation or show preference for those expressed only in particular stages. To identify genes particularly involved in early or late stages of neurodifferentiation, we retrieved high-throughput data from neurons generated in vitro via iPSCs differentiation, classified the cells to iPCSs, neural progenitors (NPCs) and mature neurons and defined the genes that are specifically expressed in the neuronal progenitors and in mature neurons. Five gene expression datasets were retrieved, which had been generated by diverse technological platforms and under different experimental conditions. Taking into account this inter-study variability, the samples from each dataset were assigned to one of the three neurodifferentiation stages or groups (i.e., iPSC, neuroprogenitors, mature neurons) based on the classification provided by the supplier; in the case a clear classification was not provided (i.e., GSE110717), then we checked for the presence of validated markers for mature neurons (ENO2, GAP43, MAP2, NEFL), functional neurons (GAD1) and synaptic neurons (BSN, SYP). Cross-dataset comparison was also performed to examine the extent of similarity between the groups from different datasets. To ensure comparability among datasets, the raw RNA-Seq and microarray transcriptomic data were processed by employing a uniform pipeline (see Methods). Differential gene expression analysis was performed between neuroprogenitors and iPSC and, similarly, mature neurons versus NPCs for each dataset separately (Figure 4a).

We found that 522 genes are specifically upregulated in NPCs compared to iPCSs, 496 are upregulated in mature neurons versus NPCs and 40 genes were expressed in both NPCs and neurons as opposed to iPSCs. Cox regression analysis in PanCan revealed that 40.42% of the NPC-specific genes, 41.73% of the neuron-specific genes and 45% of the genes expressed in both stages of the neuronal lineage are associated with poor patient outcomes, while less genes are associated with favorable outcomes (31.23%, 28.83% and 20%, respectively) (Figure 4b). Interestingly, several of these genes are also involved in neuronal development and neurological function, as indicated by their overlaps with neu.dev and behavioral genes (Figure 4c and Table S7). These include genes where the expression of which should be restricted to the brain and/or peripheral nervous system (PNS), based on data mined in the Tiger database [26]. Examples are synapsin (SYN1), which regulates neurotransmitter release at synapses [27]; neurofilaments medium and light polypeptides (NEFL and NEFM, respectively), which are particularly abundant in axons and essential for the radial growth of axons during development, the maintenance of axon caliber and the transmission of electrical impulses along axons [28]; neuregulin 3 (NRG3), a member of the neuregulin family which promotes excitatory synapse formation on hippocampal interneurons [29]; and ELAVL3, a member of the Elavl family of proteins, which bind to RNA and are crucial for the maintenance of axonal homeostasis in mature neurons [30] (Table S7). Our analysis shows that genes that are activated across several stages of neurodifferentiation are co-opted by cancer cells, and these events are more often associated with poor rather than favorable outcomes. The off-context activation of neurodifferentiation genes where the expression of which should only be restricted in the brain or PNS seems to be advantageous for tumor progression.

### 2.6. Neurogenesis and Neoneurogenesis Mediators Are Abundantly Expressed during Melanoma Progression

Tumors may provoke neoneurogenesis and induce their own innervation by secreting neuronal growth factors and axon guidance molecules. Such secreted factors could be well-established players of neurogenesis, like the nerve growth factor (NGF) and its cognate receptor (NGFR), which are usually produced by tumors, exert autocrine or paracrine effects and correlate with adverse outcomes [14]. Interestingly, the brain-derived neurotrophic factor (BDNF), a protein with brain-restricted expression, has been recently established as a marker of neoneurogenesis. In response to adrenaline, tumor cells produce and secrete BDNF in the TME, a fact that leads to tumor innervation by signaling through host neurotrophic receptor tyrosine kinase 2 receptors. In patients with cancer, high tumor nerve counts were significantly associated with increased BDNF and norepinephrine levels and decreased overall survival [31]. Notably, BDNF was identified among neu.dev and behavioral genes (Tables S1 and S2), while its increased levels are associated with disease progression in the PanCan patient cohort (Table S5).

**Figure 5:**
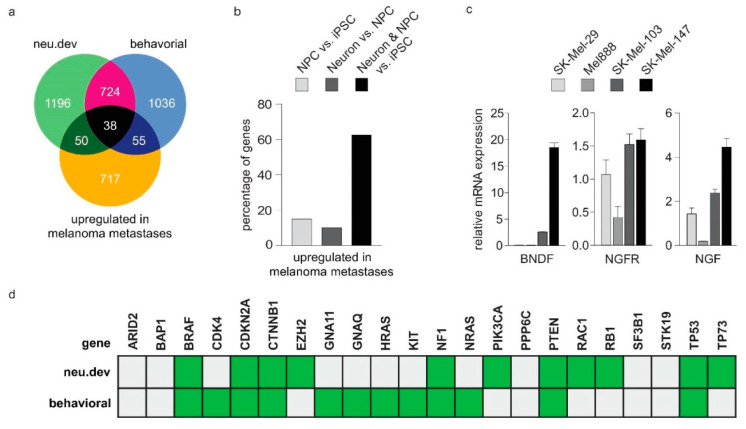
Melanoma progression is associated with upregulation of neuronal factors. (**a**,**b**) Nervous system-related genes (**a**) and genes involved in early (NPC) and late stages (neurons) of neuronal differentiation of iPSCs (**b**) are upregulated in metastatic melanoma versus primary lesions. (**c**) Neurogenesis and neoneurogenesis factors are upregulated in highly invasive SK-Mel-103 and SK-Mel-147 versus the less invasive melanoma cell lines SK-Mel-29 and Mel-888. (**d**) The majority of well-established melanoma drivers [32] and inducers of metastasis initiation [33] are indispensable for neuronal development and/or neurological function.

It is currently unknown whether associations exist between stemness and neoneurogenesis in the context of metastasis. To address this issue, we considered malignant melanoma as a representative tumor where cancer stemness has a major impact on disease progression and relapse. A recent case report of a patient presenting a melanoma lesion with cytological characteristics of nerve cells [16] provides clinical hints for neoneurogenesis in this cancer type. Moreover, by meta-analyzing transcriptomic data of metastases from TCGA melanoma patients, we found that genes upregulated in metastatic relative to primary lesions are essential for neuronal development (neu.dev), neurological function (behavioral) or both (Figure 5a). Moreover, genes commonly expressed in both NPC and neurons were found particularly upregulated in metastatic melanoma lesions (62.5%, 25 out of 40 common genes), while upregulations of NPC-specific genes (14.94%, 78 out of 522) and neuron-specific genes (10.08%, 50 out of 496) were also observed (Figure 5b), indicating that melanoma metastases express genes involved both in early and late stages of neuronal differentiation. Overall, our data strongly suggest activation of pathways mediating neuronal development, differentiation and activity during melanoma progression. With these in mind, we searched for molecular signs of neoneurogenesis in melanoma by estimating the levels of NGF, NGFR and BDNF, in conjunction with invasive phenotypes. We used a melanoma tissue culture model consisting of distinct cell lines with less or more invasive characteristics. Our data show that endogenous levels of all abovementioned neurotrophic factors are higher in more invasive SK-Mel-103 and SK-Mel-147 melanoma cell lines, but lower in less invasive SK-Mel-29 and Mel-888 (Figure 5c). Collectively, these results give us a first hint for tumor innervation during melanoma progression.

### 2.7. Neurogenesis, Neoneurogenesis and Stemness Factors Are Concomitantly Regulated by TP73-Derived Isoforms

**Figure 6:**
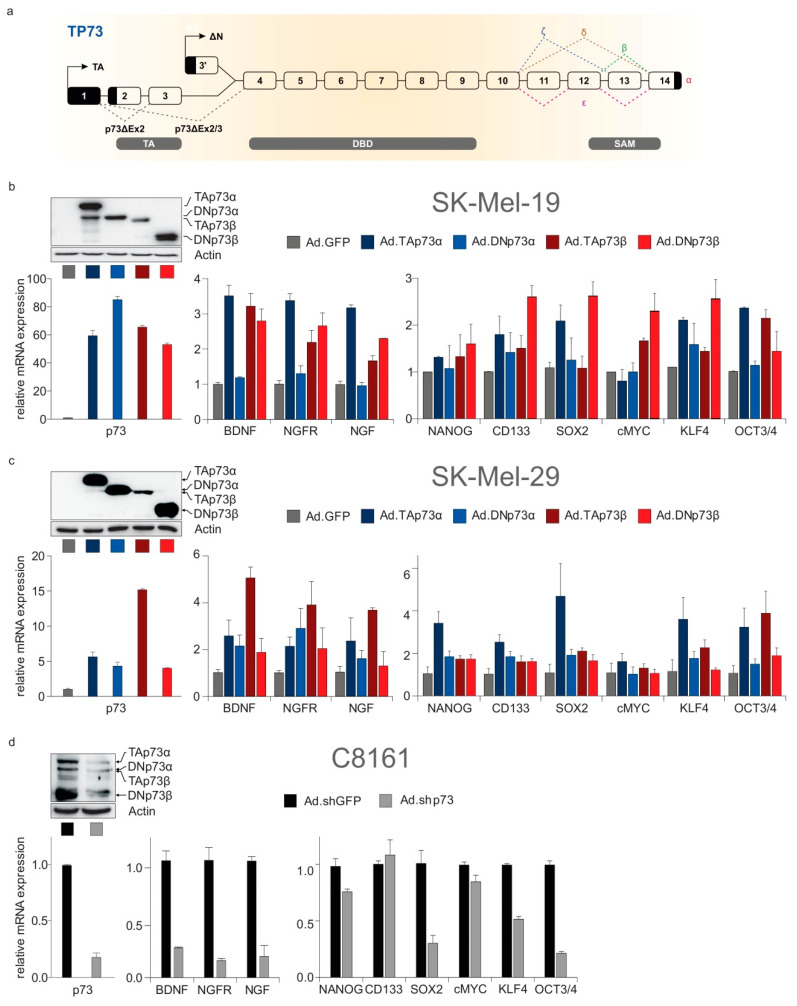
Products of the TP73 gene can simultaneously upregulate crucial stemness and neurogenesis mediators in melanoma. (**a**) Diagram depicting synthesis of isoforms from the TP73 gene. The p73 isoforms are produced by (i) alternative splicing in the 3′ end, putting forth several C-terminal splice variants (designated α, β, γ, δ, ε, ζ); or (ii) the use of an extrinsic (P1) and alternative intrinsic promoter (P2) in the 5′ end, generating TA and ΔΝ classes of isoforms, and (iii) alternative splicing in the 5′ end, resulting in truncated ΔΤA isoforms that partially or entirely lack the transactivation domain and, together with ΔΝ, constitute the DN isoforms. All isoforms contain a core DNA-binding domain. Different combinations of the N-terminal head with the C-terminal tail give rise to functionally distinct isoforms [34,36]. TA: transactivation domain, DBD: DNA-binding domain, SAM: sterile alpha motif. Melanoma progression is characterized by overexpression of the P1-derived p73ΔEx2/3 isoforms along with TAp73α. (**b**,**c**) Sufficient transduction of the low-invasive, low-p73 expressing cell lines SK-Mel-19 (**b**) and SK-Mel-29 (**c**) with TAp73 or p73ΔEx2/3 isoforms (herein labeled as DNp73) is depicted by increased p73 protein (upper panel) and mRNA (left panel) levels relative to the GFP-transduced controls. The mRNA levels of neurotrophic (mid panel) and stemness factors (right panel) upon transduction of low-invasive, low-p73 expressing cell lines with adenovirus constructs expressing distinct p73 isoforms. (**d**) Same as (**b**,**c**) for the shRNA knockdown of p73 isoforms in the high-invasive, high-p73 expressing C8161.

Based on these findings, we wondered whether melanoma driver genes mediate their aggressive effects, at least in part, by systemically activating nervous system-related genes which could, in turn, support a neurogenic phenotype. Genetic alterations in these master regulators may plausibly lead to ectopic activation of nervous system-related target genes and to reprogramming of cancer cells into neural-like cells, which could, overall, increase the metastatic potential. Strikingly, we observed that 16 out of the 21 (76.2%) well-established melanoma driver genes [32] (Figure 5d) are indispensable for neuronal development or neurological function. These include, for instance, the frequently mutated BRAF, NRAS and TP53. In the neu.dev gene group, we also found TP73, a structural and functional homologue of TP53 and the primitive member of the p53 transcription factor family [34]. While the TP73 gene itself is rarely mutated in cancer, it exerts its oncogenic activities by abundant expression of distinct isoforms (described in Figure 6a). Of interest here is that TP73-derived isoforms were identified as key players of metastasis initiation and stemness [33,35]. We have previously shown that tumor-specific DNp73 (namely, p73ΔEx2/3β) is particularly upregulated in melanoma, driving invasion via induction of EMT, whereas levels of TAp73α are also persistently high in aggressive stages [33,35]. Taking advantage of our previous work and experimental model [33,35,36,37,38], we proceeded to test the hypothesis that TP73 gene products are also involved in the cancer-neuronal crosstalk as upstream regulators of nervous system-related genes. 

The patterns of co-elevation of TAp73α and p73ΔEx2/3 isoforms with major neurogenesis factors in highly versus less invasive melanoma cells (Figure 5c) prompted us to speculate that the latter are reactivated by p73. When we transduced low-invasive melanoma cell lines with p73ΔEx2/3β and subjected the cells to transcriptome analysis, we noticed upregulation of 20 out of 176 nervous system-related genes that were in silico identified as potential indicators of neuronal co-option during cancer progression (Table S6). This coherent reactivation of several of these markers in response to p73ΔEx2/3β (Table S8) basically underscores its key upstream role in this process. Indeed, addition of TAp73α, TAp73β, p73ΔEx2/3α or p73ΔEx2/3β in low-p73 expressing, less invasive SK-Mel-19 and SK-Mel-29 causes upregulation of the neurogenesis mediators NGF, NGFR and BDNF (Figure 6b,c, mid panels). Vice versa, shRNA-mediated downregulation of all p73 isoforms in p73-overexpressing, highly invasive C8161 cells resulted in downregulation of these factors (Figure 6d, mid panel). Importantly, these genes were found to bear functional p73-responsive elements (data not shown). In parallel, stemness markers are upregulated in response to p73 isoforms (Figure 6b,d, right panels). This suggests that p73 isoforms coordinatively regulate neuro-differentiation and stemness markers in invasive tumor stages.

## 3. Discussion

Stemness is a cellular fate that enables cells to maintain pluripotency, be self-renewed and regenerate tissues. Stemness is active during ontogenesis, where embryonic stem cells are differentiated to diverse cell types, and postnatally in some differentiated tissues which contain a small percentage of somatic stem cells [39] that remain in a dynamic equilibrium with the differentiated tissue cells [39]. According to current notions, adult stem cells can be abundant in their niches and actively divide throughout life, while the fate of the stem cell progeny depends on the available niche space. Their daughter cells compete to occupy the niche in a process known as neutral competition, which leads to an extensive plasticity of stem cell hierarchies. This means that daughter cells and fully differentiated cells can re-enter the niche and dedifferentiate to replace lost stem cells. This plasticity between non-stem cells and stem cells present in normal tissues is recapitulated in cancer [40]. A solid tumor presents significant heterogeneity, which is sustained by a pluripotent cell subpopulation within the tumor, the so-called cancer stem cells (CSCs), which are frequently more enriched in metastasis-prone or metastatic tumors [35]. A bidirectional conversion is established between CSCs and non-CSCs, whereby CSCs can give rise to differentiated offspring, and conversely, any differentiated tumor cell, regardless of its differentiation state, has the potential to get dedifferentiated to a CSC, given that an appropriate stimulus induces this plasticity [40]. Notably, this CSC/non-CSC versatility is developed, at least in part, by co-opting nodal embryonic developmental programs including, but not limited to, EMT [39]. 

One question that is subsequently raised because of these recent updates of the CSC concept [40] is whether stemness is, at the same time, accompanied by modulation of differentiation programs. Our study provides in silico evidence that aggressive tumors present a bias for differentiation across the neuronal cell lineage by off-context activation of genes essential for neuronal development and function, and this process occurs in a universal rather than a cancer type-specific manner. We identify a panel of nervous system-related genes that are frequently reactivated in many cancer types in correlation with poor prognosis. Combinations of such genes could be evaluated as signature markers for a possible shift in the CSC/non-CSC equilibrium towards neurodifferentiation and be considered as predictors of neuronal co-options during metastatic dissemination. It would be interesting if neurodifferentiation and stemness pathways are co-activated in the CSCs or if there is a constant interplay between subpopulations which remain pluripotent within the CSC niche and cells outside the niche which get committed to neuronal fates, perhaps to provide selective advantages during cancer progression. From the therapeutic point of view, interference with the mechanisms that control the dynamic plasticity of CSCs and their phenotypic transitions across the neuronal differentiation lineage may provide a basis for developing next-generation therapeutic strategies against cancer stemness. Instead of targeting the CSC population itself, which could result in the dedifferentiation of surrounding differentiated cells to surrogate for the lost CSCs [40], it might be more expedient to address the mechanisms that control the cancer stemness/neurodifferentiation balance.

We further demonstrate that melanoma is a representative case where late-stage stemness is orchestrated by the activation of neurodifferentiation programs during progression to invasive stages. This was manifested by the upregulation, in aggressive melanoma states, of key neurotrophic factors, including BDNF, which was recently shown to foster neoneurogenesis. Importantly, both neurotrophins and stemness markers are responsive to p73, a major transcription factor with a dual role in cancer and in the neuronal system, as evidenced by coexistence of cancer-related phenotypes and neurodevelopmental defects and in TP73 knockout mice [31]. The way TP73 exerts these effects appears to be highly isoform-dependent [41]. Recent studies imply that p73 isoforms differing not only in their N-terminus but also in their carboxy-terminal sequences have divergent effects on neuronal differentiation, maintenance of NSCs, neuronal survival during development and in adult neuronal tissues [41]. For instance, pan-TP73KO mice display severe hydrocephalus and hippocampal dysgenesis, with loss of the lower blade of the dentate gyrus (DG) and an impaired organization of CA1 and CA3 regions. TAp73KO mice show a less severe phenotype, but still exert abnormal hippocampal development, whereas ΔNp73 mice demonstrate only marginal reduction in cortical thickness but no hydrocephalus [42]. The recently described Trp73^d13/d13^ mice, which produce a switch of the longest and most expressed isoform α into β, lead to hippocampal dysgenesis, such as progressive depauperation of Cajal–Retzius (CR) cells in the developing brain, as a consequence of deprivation of CR cells that are physiologically deputed to direct brain architecture during embryonic development [43]. In view of these data, our results suggest that p73 isoforms co-regulate stemness and neurodifferentiation to control tumor progression. This is also the first time to demonstrate the ability of the tumor-specific ΔTAp73 (p73ΔEx2/3 α and β) isoforms, which are established metastasis inducers and CSC regulators, to activate key neurodifferentiation players. The fact that the TAp73α isoform, which is traditionally considered an apoptotic isoform, has a strong effect in co-elevation of stemness and neurotrophic factors might imply a switch of its antioncogenic duties in late tumor stages, in accordance with p73ΔEx2/3 with which it is co-expressed. It would be of great interest to unveil both the underlying mechanism and the contribution of individual p73 isoforms in this process, including certain TAp73 isoforms with persistent overexpression in aggressive stages, for which recent evidence implies a Janus behavior in the context of metastasis-supporting processes [44,45]. An appealing question that warrants further investigation, in view of the complexity of p73 expression patterns in invasive stages, is whether the neurogenic phenotypes of cancer cells reflect a sophisticated combination of the specialized neuronal tasks of each p73 isoform rather than a simplistic dominance of the traditionally oncogenic DNs over the apoptotic TAs.

Our results imply that p73 isoforms may exert their documented roles in cancer invasion and metastasis (for a review, see [34,37,38]), at least partly, through manifesting their neuronal attributes and activating their nervous system-related gene targets within the cancer cell context. Indeed, increasing hints support co-option of p73-mediated neuronal pathways in the context of tumor progression. For example, TAp73 isoforms control cancer cell proliferation, migration and invasion through transactivation of the brain-enriched miRNA gene MIR3158, which targets vimentin [46]. Similarly, p73ΔEx2/3 expression in less invasive melanoma cells enhances stemness and self-renewal capacity through an interplay with MIR885 [35], an miRNA with brain/cerebellum-restricted expression (data mined from miRiad database [47]), that targets IGFR [35]. Again in an IGFR-dependent manner, p73ΔEx2/3 drives EMT phenotypic conversion and initiation of metastasis [33], along with tyrosinase degradation, depigmentation and loss of melanocyte identity [48]. In the presence of p73ΔEx2/3 and persistently high TAp73α levels, melanoma cells lose their original cell-type characteristics and simultaneously activate stemness [35], EMT [33] and nervous system-related genes (this study). The increased cancer stemness, along with loss of original cell identity (dedifferentiation) and acquisition of characteristics of neuronal cell types, is overall indicative of switching identity. 

An appealing hypothesis that warrants future investigation is that, by activating neuronal networks, the cancer cells might impersonate neurons to summon neuronal cell progenitors in the TME and develop complex interactions which sustain a prometastatic microenvironment or provide overall selective advantages for tumor evolution. The production of neuronal signaling molecules by cancer cells could plausibly affect the behavior of adjacent neuronal cells and modify the dynamics of the complex cellular interactions within the TME. In this respect, the paradigm of p73-driven melanoma progression could represent a suitable model to mechanistically address stemness and neurodifferentiation and untangle how their interplay influences the TME and the behavior of surrounding neurons.

Last but certainly not least, the factors released by cancer cells to the TME force surrounding cells to integrate into the stroma where their activities are redirected to benefit tumor cells. Affected cells include fibroblasts, endothelial cells, bone marrow-derived cells, immune cells and neuronal extensions. It has been shown that not only immune, but also endothelial and neuronal cells express receptors that respond to oncogenic cues. Reciprocal communication between immune and nervous systems correlates with unfavorable prognosis [49] and it is highly likely that in the TME, complex reciprocal interactions between cancer cells, immune cells and neuronal cells may take place. Due to our data, it would be beneficial to extend the melanoma cell–immune cell interactions to also include neuronal cells and to investigate this cancer–neuro–immune crosstalk in the TME. Neurotrophins such as NGF and BDNF [50,51] represent attractive signaling molecules that could act as common denominators for such complex cell–cell interactions and are recognizable by all three cell types. For instance, the hypothesis that cancer cells may recruit neuronal cells to take advantage of the ability of the CNS to control inflammation in terms of immune privilege could be tested. This would then allow cancer cells to dictate the fate of the immune cells in the TME to their benefit, depending on the changes in the microenvironment milieu. Addressing these issues can provide the foundation for elucidating the mechanisms of the nerve–tumor cell crosstalk in metastasis and the development of antineurogenic cancer therapeutics.

## 4. Materials and Methods 

### 4.1. Cell Culture and Treatments

SK-Mel-19, Mel-888, SK-Mel-29, C8161, SK-Mel-103 and SK-Mel-147 cells were cultured and maintained as previously described [33].

### 4.2. Adenovector Generation and Transduction

Ad.TAp73β and Ad.DNp73β vectors have been described earlier [33]. For the construction of Ad.TAp73α and Ad.DNp73α, the sequences were excised from pcDNA3.1 plasmids [52] using NheI and HindIII and after, they were Klenow treatment-cloned into EcoRV-linearized pAdTrack-CMV. All constructs were verified by sequencing analysis (Sequence Laboratories Göttingen). The virus was generated by homologous recombination following transformation with pAdEasy1 containing BJ5183. All viruses were propagated, purified and titrated as described. Ad.TAp73β, Ad.DNp73β, Ad.shGFP and Ad.shp73 were generated as described previously [33,35]. SK-Mel-19 and SK-Mel-29 cells were transiently transduced with adenovectors Ad.GFP, Ad.TAp73α, Ad.DNp73α, Ad.TAp73β and Ad.DNp73β at MOI 2.5. Transduction of C8161 was performed with Ad.sh.GFP and Ad.sh.p73 at MOI 30.

### 4.3. RNA Isolation and q-PCR

After cell harvest RNA isolation, reverse transcription and qPCR were performed as described using GAPDH or β-actin for normalization. The primer sequences are presented in Table S9.

### 4.4. Western Blot

Cell protein lysates were produced and immunoblotting was conducted as described previously using antibodies against p73 (ER15) (BD Biosciences) and β-actin (Sigma). 

### 4.5. Identification of Nervous System-Related Genes 

To identify genes that are indispensable for nervous system development and function, we screened the Mouse Genome Informatics database for candidates whose knockout leads to mouse phenotypes with neurodevelopmental, neurological and/or behavioral defects. To this end, we downloaded the “Mammalian Phenotype (MP)-Mouse Developmental Anatomy (EMAPA) Mappings” and the “All Genotypes and Mammalian Phenotype Annotations (tab-delimited)” files from MGI. For comprehensive identification of the neu.dev gene list, we filtered both files using terms for anatomical regions of the nervous system and for neuronal cell processes, in order to retrieve a list of relevant mouse phenotype IDs (MP ID). The list of neu.dev-related MP IDs was used as a query in the field “Phenotype/Disease” of “Phenotypes and mutant alleles option” from “Phenotypes, Alleles & Disease Models Query”. The resulting gene list was reciprocally used as input in the “Batch query” option, in order to match each gene with the corresponding nervous system-related MP IDs and phenotype descriptions. Since the EMAPA file contains IDs for mouse developmental anatomies which correspond to specific MP IDs, this approach enabled us to obtain information on genes, their corresponding mouse phenotypes and the anatomical part which is affected by knockout of these genes. For the behavioral list, we filtered the “All Genotypes and Mammalian Phenotype Annotations (tab-delimited)” files with terms for neurological and behavioral function to retrieve a list of relevant mouse phenotype IDs (MP ID). Based on these MP IDs, we generated a list with behavioral genes and their corresponding mouse phenotypes, using the abovementioned procedure. Then, human orthologues of these genes were identified and the official HGNC [53] gene symbols were used. The comprehensive lists of neu.dev and behavioral genes are depicted in Tables S1 and S2. 

### 4.6. Gene Expression Analysis in CCLE Database

For analysis of the CCLE (Cancer Cell Line Encyclopedia), the RNA-Seq raw count datasets were downloaded from Broad Institute (Cambridge, MA, USA) [21]. Raw read counts were converted to counts per million (CPM) using the *edgeR* package in R, and genes with CPM < 0.5, as well as genes expressed in less than 100 of the cell lines, were discarded. The CPM values were processed using voom from the *limma* package and merged with the aggressive-state signature for each cell line based on the expression ratio of E-cadherin versus the sum of N-cadherin, vimentin, ZEB1 and SNAI1. Differentially expressed genes in 16 CCLE tissues (biliary tract, breast, central nervous system, endometrium, hematopoietic and lymphoid tissue, kidney, lung, ovary, pancreas, pleura, skin, soft tissue, stomach, thyroid, upper aerodigestive tract and urinary tract) were detected, pooled and compared with the list of neu.dev or behavioral genes, as well as with the random gene lists using R. The expression correlation matrix and the median expression correlation (MEC) for each gene in each list were calculated using Spearman correlation in R.

### 4.7. Kaplan–Meier Survival Analysis of TCGA Patient Data

Gene expression data (GDC-PANCAN.htseq_fpkm-uq.tsv.gz) and survival data (GDC_TCGA_PANCAN_patient_pheno_surv.txt) from the TCGA PanCan cohort [23] were downloaded from the UCSC Xena browser (https://xenabrowser.net/datapages/), filtered for up to ten-year follow-up data (3650 days) and juxtaposed with the respective gene lists (neu.dev, behavioral, random). Unless stated otherwise, all plots and statistical tests were generated using Graph Pad Prism (San Diego, CA, USA).

### 4.8. Neurodifferentiation Gene Expression Data Analysis

#### 4.8.1. Transcriptomic Data Retrieval

The public repository NCBI GEO (Gene Expression Omnibus) DataSets [54] and ArrayExpress [55,56] were searched extensively for gene expression datasets using relevant keywords: (“induced pluripotent stem cells” or “iPSC”) and (“neuron” or “neural cells”) and (“neural progenitor cells” or “neuroprogenitor” or “NPC”) and (“glia” or “glial cells” or “neuroglia”) and (“human” or “homo sapiens”). A total of five eligible datasets were selected (Table S10): The GEO series GSE74358 [57] includes global gene expression profiling by microarray of (i) undifferentiated iPSCs, (ii) neuroprogenitors (NP) and (iii) neurons at the early or late stage of differentiation. The Affymetrix human genome U133 Plus 2.0 Array platform was employed. In GSE110717 [58], genome-wide gene expression by RNA-Seq was performed for (i) iPSC and (ii) iPSC-differentiated midbrain NP. The sequencing platform Illumina HiSeq 1000 was used. In GSE65106 [59], global transcriptome profiling by microarray was performed for (i) undifferentiated iPSCs, (ii) neuroprogenitor cells (NPC) and (iii) mature neurons. The Affymetrix Human Gene 1.0 ST Array platform was employed. The GEO series GSE135287 [60] and GSE124308 include genome-wide gene expression analysis by RNA-Seq of human iPSC-derived cortical (i) neural progenitor cells and (ii) mature neurons. The sequencing platforms Illumina NextSeq 500 and Illumina HiSeq 2000 were employed.

#### 4.8.2. Differential Gene Expression Analysis

For microarray data, GEO2R [54], an R-based interactive web application, was employed for the detection of differentially expressed genes (DEG) in the microarray datasets GSE74358 and GSE65106. The distribution of gene expression values for the samples was investigated, setting the threshold values for absolute log fold change ∣logFC∣ ≥ 2 and FDR-adjusted *p*-value ≤ 0.05. For RNA-Seq data, the raw read counts for each sample per dataset (GSE110717, GSE135287, GSE124308) were downloaded from GEO, by using bash scripting. All calculations and statistics for RNASeq data were performed by using the R v4.0.2 software environment (https://cran.r-project.org/). Genes with a count per million (CPM) greater than 0.5 in at least two samples were selected. The *edgeR* package (version 3.30.3) was employed to perform differential gene expression analysis, setting the cutoff values ∣logFC∣ ≥ 2 and FDR-adjusted *p*-value ≤ 0.05. The *pheatmap* package v. 1.0.12 was used to create heatmaps of DEGs. Expression of commercially available markers of the neural lineage (https://www.abcam.com/neuroscience/neural-markers-guide) was used for validation of the stage of differentiation and the classification of samples to iPSCs, NPCs or mature neurons.

### 4.9. Statistical Analysis

A statistical analysis was performed using the z-test. *P* values less than 0.05 were considered as significant. * *p* < 0.05. ** *p* < 0.01. *** *p* < 0.001. **** *p* < 0.0001.

## 5. Conclusions

By applying computational approaches, combined with validated experimental results, we provide compelling evidence that reactivations of genes involved in neuronal development, neuronal cell differentiation and neurological function within the tumor context drive disease progression. Upregulations of genes that are indispensable for nervous system development and/or behavior in the tumor context correlate with poor patient survival. Activation of neurotrophic factors, including the recently identified neoneurogenesis mediator BDNF, is evident during melanoma progression. In melanoma cells, the progression of which is driven by DNp73, neurodifferentiation pathways are activated in conjunction with stemness markers. In this study, we systemically investigated the neuronal processes that are co-opted across cancer types to enhance metastatic potential and highlighted the p73-regulated networks as emerging players of cancer neurobiology.

## Supplementary Materials

The following are available online at https://www.mdpi.com/2072-6694/12/12/3789/s1, Tables S1–S9. Table S1: Mouse gene knockouts which present abnormalities in neuronal development. Table S2: Mouse gene knockouts which present abnormalities in neurological/behavioral function. Table S3: 100 lists of random coding genes as controls for neu.dev (upper) and behavioral (lower) genes. Table S4: CCLE classification, cancer cell lines classified into less and highly invasive based on the EMT markers expression profiles. Table S5: Nervous system-related genes and their effect on patient survival prognosis in PanCancer based on Cox regression analysis. Table S6: Nervous system-related genes and their effect on patient survival prognosis in different cancer types of TCGA based on Cox regression analysis. Table S7: Poor prognostic factors with brain or peripheral nervous system-specific expression profiles. Table S8: Nervous system-related genes that predict poor prognosis across 10–17 cancer types, and their responsiveness to prometastatic p73ΔEx2/3β. The p73ΔEx2/3β-responsive predictors are highlighted in red. Table S9: Primers used in qPCR. Table S10: Samples per gene expression dataset analyzed in the present study. The samples indicated by red were compared to those shown in blue.
