# Supplementary figures and images for: Neural Networks Recapitulation by Cancer Cells Promotes Disease Progression: A Novel Role of p73 Isoforms in Cancer-Neuronal Crosstalk
Source: Cancers (Basel). 2020 Dec 16;12(12):3789. doi: 10.3390/cancers12123789 (PMC7765507; doi:10.3390/cancers12123789)

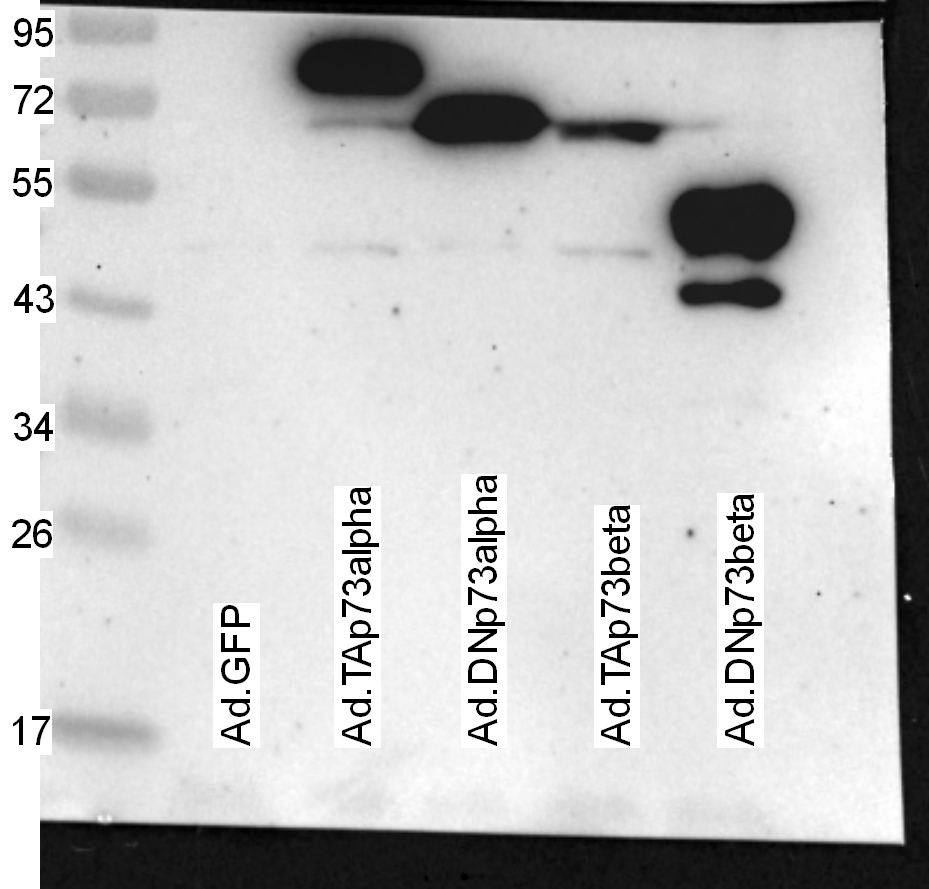

Supplement: Supplementary file 1 [file cancers-12-03789-s001.zip › cancers-1034553-supplementary materials/Fig. 5e/1_WB_p73_isoforms,overlap.tif]

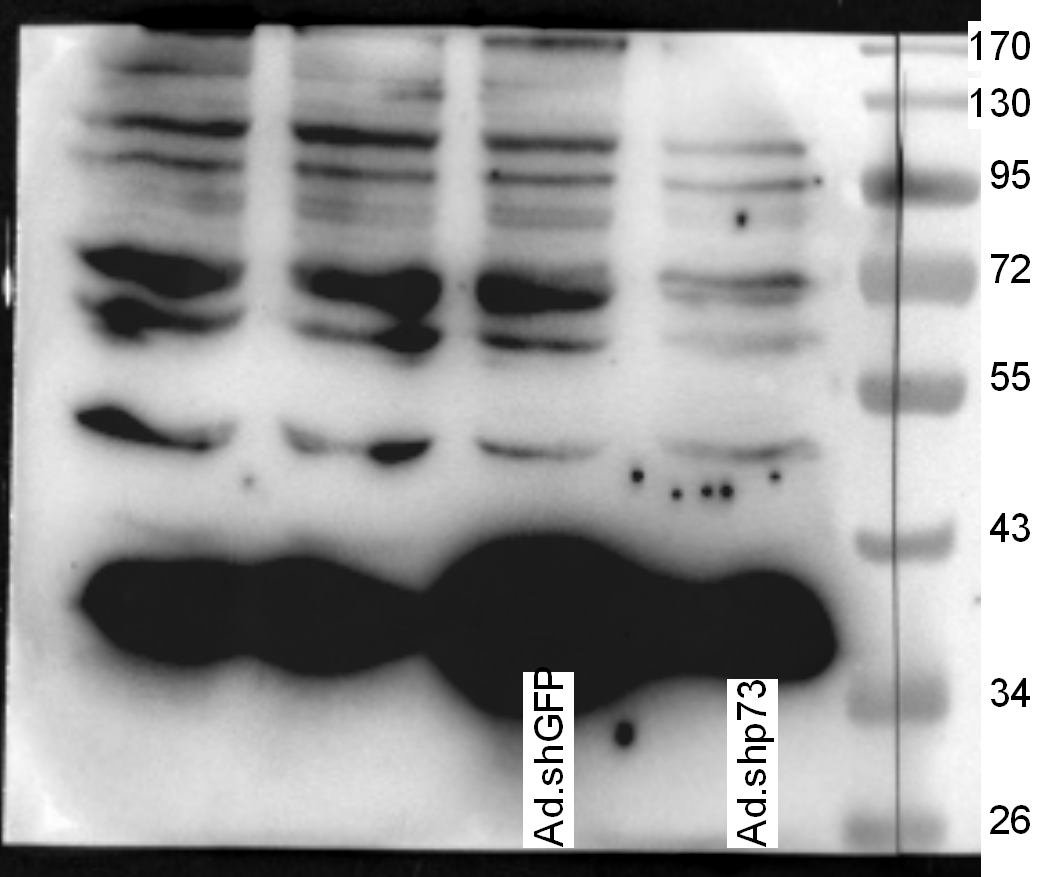

Supplement: Supplementary file 1 [file cancers-12-03789-s001.zip › cancers-1034553-supplementary materials/Fig. 5f/2_WB_shp73_KD,overlap.tif]
